# Adjunctive dexamethasone for the treatment of HIV-uninfected adults with tuberculous meningitis stratified by Leukotriene A4 hydrolase genotype (LAST ACT): Study protocol for a randomised double blind placebo controlled non-inferiority trial

**DOI:** 10.12688/wellcomeopenres.14007.1

**Published:** 2018-03-20

**Authors:** Joseph Donovan, Nguyen Hoan Phu, Le Thi Phuong Thao, Nguyen Huu Lan, Nguyen Thi Hoang Mai, Nguyen Thi Mai Trang, Nguyen Thi Thu Hiep, Tran Bao Nhu, Bui Thi Bich Hanh, Vu Thi Phuong Mai, Nguyen Duc Bang, Do Chau Giang, Dang Thi Minh Ha, Jeremy Day, Nguyen TT Thuong, Nguyen Nang Vien, Ronald B. Geskus, Tran Tinh Hien, Evelyne Kestelyn, Marcel Wolbers, Nguyen Van Vinh Chau, Guy E. Thwaites

**Affiliations:** 1 Oxford University Clinical Research Unit, Ho Chi Minh City, Vietnam; 2 Centre for Tropical Medicine and Global Health, Nuffield Department of Medicine, University of Oxford, Oxford, UK; 3 Hospital for Tropical Diseases, Ho Chi Minh City, Vietnam; 4 Pham Ngoc Thach Hospital, Ho Chi Minh City, Vietnam

**Keywords:** Tuberculous meningitis, HIV-uninfected, Dexamethasone, Drug induced liver injury, LTA4H, Adrenal suppression, Hyponatraemia

## Abstract

**Background: **Tuberculosis kills more people than any other bacterial infection worldwide. In tuberculous meningitis (TBM), a common functional promoter variant (C/T transition) in the gene encoding leukotriene A4 hydrolase (LTA4H), predicts pre-treatment inflammatory phenotype and response to dexamethasone in HIV-uninfected individuals. The primary aim of this study is to determine whether LTA4H genotype determines benefit or harm from adjunctive dexamethasone in HIV-uninfected Vietnamese adults with TBM. The secondary aim is to investigate alternative management strategies in individuals who develop drug induced liver injury (DILI) that will enable the safe continuation of rifampicin and isoniazid therapy.

**Methods: **We will perform a parallel group, randomised (1:1), double blind, placebo-controlled,  multi-centre Phase III non-inferiority trial, comparing dexamethasone versus placebo for 6-8 weeks in addition to standard anti-tuberculosis treatment in HIV-uninfected patients with TBM stratified by LTA4H genotype. The primary endpoint will be death or new neurological event. The trial will enrol approximately 720 HIV-uninfected adults with a clinical diagnosis of TBM, from two hospitals in Ho Chi Minh City, Vietnam. 640 participants with CC or CT- LTA4H genotype will be randomised to either dexamethasone or placebo, and the remaining TT- genotype participants will be treated with standard-of-care dexamethasone. We will also perform a randomised comparison of three management strategies for anti-tuberculosis DILI. An identical ancillary study will also be perfomed in the linked randomised controlled trial of dexamethasone in HIV-infected adults with TBM (ACT HIV).

**Discussion: **Previous data have shown that LTA4H genotype may be a critical determinant of inflammation and consequently of adjunctive anti-inflammatory treatment response in TBM. We will stratify dexamethasone therapy according to LTA4H genotype in HIV-uninfected adults, which may indicate a role for targeted anti-inflammatory therapy according to variation in LTA4H C/T transition. A comparison of DILI management strategies may allow the safe continuation of rifampicin and isoniazid.

## Introduction

### Background

Tuberculous meningitis (TBM) is the most severe form of tuberculosis, resulting in death or neurological disability in approximately 40% of HIV-uninfected sufferers, and 70% of those co-infected with HIV. Effective anti-tuberculosis chemotherapy has been available for more than 50 years, yet tuberculosis kills more people than any other single bacterial infection worldwide. Diagnosing TBM is difficult and depends upon detecting low numbers of *M. tuberculosis* bacteria in cerebrospinal fluid (CSF). Rifampicin (R), isoniazid (H), pyrazinamide (Z) and ethambutol (E) are the first-line antibiotics for all forms of drug-susceptible tuberculosis. These agents, used daily and in combination, kill the bacteria and prevent the emergence of resistance. There is concern that the blood-brain barrier reduces the concentrations of anti-tuberculosis drugs in the brain which may attenuate both bacterial killing and clinical response ^1^. The most recent Cochrane meta-analysis and systematic review of adjunctive corticosteroids for TBM ^2^ concluded that corticosteroids likely reduced death from TBM in HIV-uninfected individuals in the short term (up to 18 months of follow-up), but had no clear impact on longer term neurological disability or survival.

### Complications of TBM

Hydrocephalus, stroke, and tuberculoma formation are important and common complications of TBM ^3^, however little evidence exists to guide their optimal management. Drug induced hepatitis associated with anti-tuberculosis treatment occurs in around 10% of HIV-uninfected patients ^4^, and can be attributed to rifampicin, isoniazid or pyrazinamide. Determining which drug is responsible is difficult. Corticosteroid therapy leads to hyperglycaemia, and during treatment of TBM dexamethasone may worsen, or reveal, diabetes. Severe hyponatraemia (low plasma sodium), high temperature and CO _2_ retention all exacerbate raised intracranial pressure and require active management. The role of corticosteroids in adrenal suppression in TBM requires further investigation.

### The role of LTA4H

How corticosteroids improve survival in HIV-uninfected patients with TBM, and whether they do so in all HIV-uninfected patients, remains uncertain and is the focus of the LAST ACT trial. A common functional promoter variant (C/T transition) in the gene encoding LTA4H, which determines the balance of pro- and anti-inflammatory eicosanoids, appears to predict pre-treatment inflammatory phenotype and response to dexamethasone in HIV-uninfected participants ^5, 6^. In humans, LTA4H rs17525495 allele homozygosity (TT and CC respectively) was associated with susceptibility to mycobacterial infection, albeit with opposite inflammatory states - high for the TT-genotype and low for the CC-genotype ^5^. Heterozygotes (CT) had an intermediate inflammatory response, and were more likely to survive TBM ^5^. A retrospective study, analysing HIV-uninfected Vietnamese adults with TBM enrolled into an earlier randomised controlled trial of adjunctive dexamethasone ^7^ and prospective TBM observational studies, found that the survival benefit of dexamethasone was restricted to the hyper-inflammatory LTA4H TT-genotype patients, with possible harm suggested in hypo-inflammatory CC-genotype patients ^5^. This suggests LTA4H genotype may be a critical determinant of inflammation and consequently of adjunctive anti-inflammatory treatment response.

### Hypotheses

Dexamethasone has been shown to improve survival in HIV-uninfected individuals with TBM ^2^. Previous data strongly suggests hyperinflammatory LTA4H TT-genotype patients with TBM benefit from dexamethasone, and that adjunctive dexamethasone may not benefit, and may cause harm, when given to patients with LTA4H CT or CC-genotype. **Our primary hypothesis is that dexamethasone has no benefit in patients with CC or CT genotype for adults with TBM**. We regard the benefit of dexamethasone in patients with LTA4H TT-genotype as established and will hence not randomise them. In contrast, patients with CT or CC-genotypes will be randomised in a practice-defining randomised controlled trial (RCT) to assess whether placebo is non-inferior or even superior to dexamethasone in these patients. Our secondary hypothesis is that current guidelines for the management of anti-tuberculosis DILI in those with TBM result in the premature cessation of rifampicin and isoniazid (the critical active drugs in early therapy) and place patients at unnecessary risk of TBM-related death and disability.

## Study aims

### Primary aim

Our primary aim is to determine whether LTA4H genotype, defined at randomisation, determines dexamethasone’s clinical effectiveness when added to the first 6–8 weeks of anti-tuberculosis treatment of TBM with dexamethasone duration depending on Medical Research Council (MRC) grade ( Supplementary File 1) at the start of treatment. In making this assessment we not only determine whether in LTA4H CT or CC-genotype patients placebo is non-inferior to dexamethasone with respect to survival and the incidence of new neurological events, but also with respect to disability assessed by the modified Rankin scale ( Table 1), the frequency of severe and serious adverse events, and the need for rescue corticosteroids.

**Table 1.** Modified Rankin scale.

Score	Description
0	No symptoms
1	Minor symptoms not interfering with lifestyle
2	Symptoms that lead to some restriction in lifestyle, but do not interfere with the patients ability to look after themselves
3	Symptoms that restrict lifestyle and prevent totally independent living
4	Symptoms that clearly prevent independent living, although the patient does not need constant care and attention
5	Totally dependent, requiring constant help day and night
6	Death

### Secondary aims

Our secondary aim is to investigate alternative management strategies in a subset of patients who develop DILI that will enable the safe continuation of rifampicin and isoniazid therapy whenever possible.

### Design and setting

**Figure 1.**
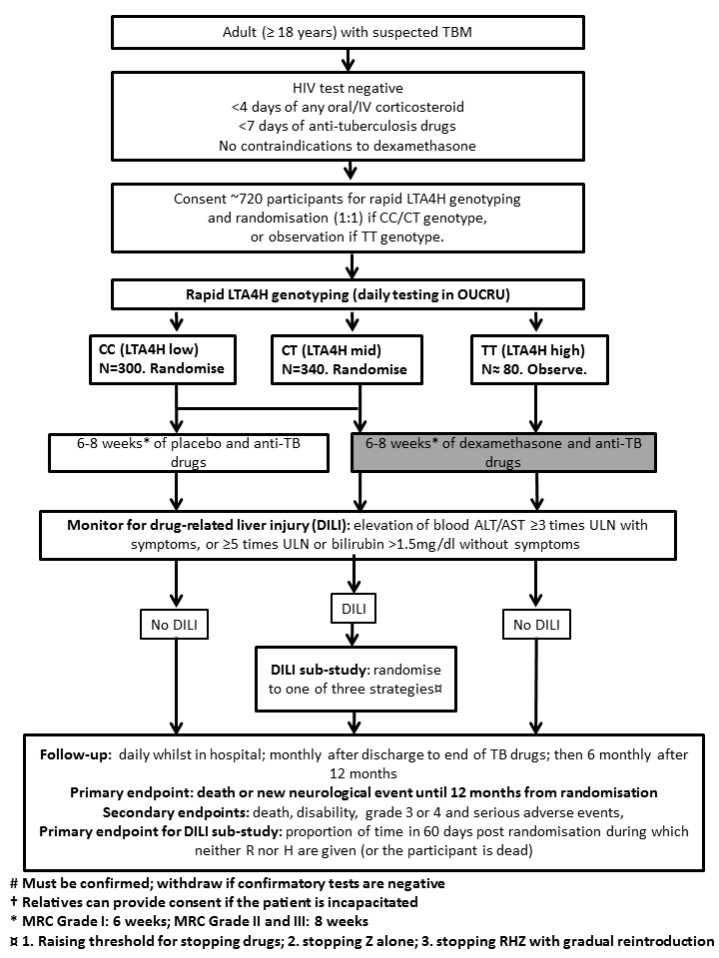
Trial Schema for LAST ACT trial (main trial).

LAST ACT is a parallel group, randomised (1:1), double blind, placebo-controlled, multi-centre Phase III non-inferiority trial, comparing dexamethasone versus placebo for 6-8 weeks in addition to standard anti-tuberculosis treatment in HIV-uninfected patients with TBM stratified by LTA4H genotype. ** The trial will be set in two hospitals in Ho Chi Minh City, Vietnam. The trial will enrol approximately 720 HIV-uninfected adults (≥18 years) with a clinical diagnosis of TBM, as judged by the attending physician, requiring immediate anti-tuberculosis treatment. Amongst these, 640 participants with CT or CC- LTA4H genotype will be randomised to either dexamethasone or placebo. The remaining participants (approximately 80) with TT-genotype will be treated with standard-of-care dexamethasone. The trial schema is shown in Figure 1.

**Figure 2.**
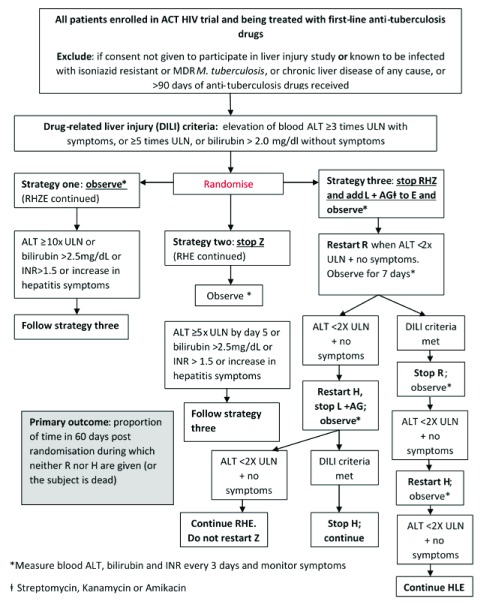
Trial Schema for DILI strategy study.

An ancillary DILI strategy study will perform a randomised comparison of management strategies in DILI early in anti-tuberculosis treatment (the intensive phase). We will perform an open, randomised comparison of three management strategies with the aim of demonstrating which strategy results in the least interruption in rifampicin and isoniazid treatment. All patients enrolled in the main trial will be eligible to take part in this study, with the exception of those known to have TBM caused by isoniazid resistant or MDR *M. tuberculosis*. An identical ancillary study will also be performed in the linked RCT of dexamethasone in HIV-infected adults with TBM (ACT HIV) ^8^. The results from both trials will be reported together, after the primary results from both trials have been published. This trial schema is shown in Figure 2.

### Ancillary studies

Seven ancillary studies will be conducted within the trial. The studies are as follows:

**Ancillary study 1:** A randomised comparison of management strategies in response to DILI (as above). (All patients.)
**Ancillary study 2:** Host and bacterial genetic determinants of treatment response. We hypothesise that the LTA4H gene, and genes involved in related inflammatory pathways, may influence participant inflammatory state, TBM severity, and treatment response. We will also examine genetic variants associated with the development of DILI. (All patients.)
**Ancillary study 3:** Impact of dexamethasone on CSF inflammation and gross cerebral pathology (assessed by serial brain MRI). We will investigate how dexamethasone influences the resolution of inflammatory markers in the CSF and the gross pathological consequences of TBM on the brain (hydrocephalus, stroke, and tuberculoma formation). (Hospital for Tropical Diseases (HTD) patients only.)
**Ancillary study 4:** Influence of diabetes mellitus on presentation and response to treatment. We will investigate whether diabetes mellitus influences clinical presentation and CSF inflammatory phenotype (linking with ancillary study 2), and how it impacts upon treatment outcomes. (All patients)
**Ancillary study 5:** Influence of Strongyloides infection on presentation and response to treatment. We will determine whether Strongyloides co-infection alters clinical and/or CSF inflammatory phenotype at TBM presentation (linking with ancillary study 2) and treatment response. (All patients.)
**Ancillary study 6:** Pathophysiology and treatment of hyponatraemia and raised intracranial pressure. We will investigate the pathophysiology of TBM-associated hyponatraemia, enabling a better understanding of the causes of hyponatraemia, the relationship between plasma sodium and elevated intracranial pressure, and the best management of severe hyponatraemia. (HTD patients only.)
**Ancillary study 7:** Dexamethasone induced adrenal suppression. We will investigate whether the use of corticosteroids in the doses prescribed in infectious diseases is associated with significant adrenal suppression. (HTD patients only.)

## Endpoints

### Primary endpoint – main trial

The primary endpoint is death or new neurological event (defined as a fall in Glasgow coma score (GCS) by ≥2 points for ≥2 days from the highest previously recorded GCS (including baseline) or the onset of any of the following clinical adverse events: cerebellar symptoms, focal neurological signs, or new seizures) during 12 months from randomisation. Survivors without a new neurological event known to be alive at 12 months will be censored at that time-point and subjects who withdrew or were lost to follow-up before 12 months will be censored at the date they were last known to be alive.

### Primary endpoint – DILI strategy study

In the DILI strategy study the primary endpoint is the proportion of time in the 60 days following randomisation during which neither rifampicin nor isoniazid are given (or the subject is dead).

### Secondary endpoints – main trial

The secondary endpoints of the main trial are as follows:

a) Overall survival during a follow-up period of 12 months. Overall survival is defined as the time from randomisation to death, during a follow-up period of 12 months. Survivors known to be alive at 12 months will be censored at that time-point and subjects who withdrew or were lost to follow-up before 12 months will be censored at the date they were last known to be alive.
b) Neurological disability at 12 months. Disability will be assessed by the modified Rankin scale ( Table 1) on days 30 and 60 from randomisation, and then monthly until the end of anti-tuberculosis drugs. The main endpoint is the 12-month assessment and subjects who died before 12 months will be treated as having a score of 6 (‘Dead’).
c) Severe (grade 3&4) and serious adverse events. Comparison of the frequency of severe and serious adverse events between treatment groups will form an important part of the study analysis.
d) Requirement for ‘rescue’ corticosteroids. Neurological complications occurring after the start of anti-tuberculosis chemotherapy for TBM are common. The cause varies, but includes hydrocephalus, infarcts, tuberculoma formation and hyponatraemia. If the symptoms are thought to be caused by tuberculomas, doctors may re-start or increase the dose of corticosteroids. In this trial, any re-start or dose increase of corticosteroids during the 12-month follow-up will be defined as ’rescue’ corticosteroids.

### Secondary endpoints – DILI strategy study

The secondary endpoints of the DILI strategy study are as follows:

a) Development of acute liver failure (defined as new onset coagulopathy (International normalised ratio (INR)>1.5 and hepatic encephalopathy) after randomisation.
b) ART interrupted due to drug-related injury (applies to patients enrolled from linked ACT HIV trial).
c) Time to new neurological event (defined as a fall in GCS of ≥2 points for ≥48 hours, new focal neurological sign, or new onset of seizures) or death from randomisation until the 12 month follow-up of the main trial.
d) Overall survival, i.e. time to death from any cause, until the 12 month follow-up of the main trial.
e) Neurological disability at the 12 month follow-up of the main trial.

## Inclusion and exclusion criteria

### Inclusion criteria

Study participants for LAST ACT must be adults (aged 18 years or older), HIV-uninfected, with a clinical diagnosis of TBM (≥5 days of meningitis symptoms, and CSF abnormalities) and anti-tuberculosis chemotherapy either planned or started by the attending physician. Participants will be considered eligible for enrolment in this trial if they fulfil all the inclusion criteria and none of the exclusion criteria. The inclusion and exclusion criteria apply at the point of consent, just before the patient’s blood is sent for LTA4H genotyping. Therefore, it is possible that when the LTA4H genotype result returns and the patient is randomised, the patient will have had >3 days of corticosteroids and/or >6 days of anti-tuberculosis treatment. This is acceptable, although the total duration of corticosteroid and anti-tuberculosis treatment before randomisation must be recorded. The time for an LTA4H result to return is not anticipated to be >3 days (e.g. taken on a Friday afternoon and processed on a Monday morning). As TBM is a serious infection requiring hospitalisation, all eligible participants will be treated in hospital, at least for the initial 3 weeks of their illness.

Study participants for the DILI strategy study must be receiving first-line anti-tuberculosis drugs and fulfil the definition of drug-related liver injury: elevation of blood transaminase concentrations ≥3 times the ULN with symptoms and signs of hepatitis (vomiting, abdominal pain, jaundice), or ≥5 times the ULN or a rise in serum bilirubin >2.0mg/dL (>34 µmol/L) without symptoms ^9^, and less than 90 days of anti-tuberculosis drugs given.

### Exclusion criteria

Exclusion criteria for the main trial are:

a) An additional brain infection (other than TBM) confirmed or suspected: positive CSF Gram or India Ink stain; positive blood or CSF Cryptococcal antigen test; cerebral toxoplasmosis suspected and attending physician wants to give anti-toxoplasmosis treatment with anti-tuberculosis treatment.
b) More than 6 consecutive days of two or more drugs active against *M. tuberculosis* immediately before screening.
c) More than 3 consecutive days of any type of orally or intravenously administered corticosteroid immediately before randomisation.
d) Dexamethasone considered mandatory for any reason by the attending physician.
e) Dexamethasone considered to be contraindicated for any reason by the attending physician.
f) Patient has previously been randomised into the trial for a prior episode of TBM.
g) Lack of consent from the participant or family member (if the participant is incapacitated by the disease).

Exclusion criteria for the DILI strategy study are:

a) TBM known to be caused by isoniazid resistant or MDR *M. tuberculosis* or standard first-line anti-tuberculosis drugs unable to be given for any reason other than DILI.
b) Signs of chronic liver disease of any cause (hepatosplenomegaly, prolonged jaundice, caput medusa, spider angiomata, ascites, oedema).
c) Lack of consent from the participant or family member (if the participant is incapacitated by the disease).
d) Elevation of blood transaminase concentrations ≥3 times the ULN with symptoms and signs of hepatitis (vomiting, abdominal pain, jaundice), or ≥5 times the ULN or a rise in serum bilirubin >2.0mg/dL (>34 µmol/L) without symptoms, at baseline (day 0).

### Recruitment, retention and randomisation

#### Recruitment and retention

Recruitment activities will only occur in an inpatient hospital setting (Hospital for Tropical Diseases and Pham Ngoc Thach Hospital, Ho Chi Minh City, Vietnam). The target sample size of around 720 participants will be enrolled within an anticipated accrual rate of 4 years. Once discharged from hospital the participants will be contacted by phone to remind them of their next visit. Patients who miss a visit will be contacted by phone for a maximum of three times after which a maximum of three home visits will be conducted. As part of routine clinical care participants with suspected TBM will have an HIV test, a lumbar puncture, and a GeneXpert MTB/RIF test on CSF. When possible, participants will be screened for eligibility on the day their CSF results return and at the time the decision is made to start anti-tuberculosis chemotherapy for suspected/confirmed TBM. For the DILI strategy study, based on enrolment of all consenting and eligible patients, we anticipate a total sample size of 170 participants (around 70 HIV-uninfected participants from the LAST ACT trial and a further 100 patients from the linked ACT HIV trial ^8^.).

#### Randomisation

Once a participant or their relative has provided written informed consent to enter the study, 5mls of blood will be taken for rapid LTA4H genotyping. The blood will be transported immediately to the OUCRU laboratory within HTD where daily LTA4H genotyping (except at weekends) will be performed. Randomisation will occur once the result of the LTA4H genotyping is available. LTA4H CC- and CT-genotype participants will be randomised to two parallel groups in a 1:1 ratio, receiving either dexamethasone or placebo for 6–8 weeks (according to disease severity). TT-genotype participants will be treated with open-label dexamethasone for 6-8 weeks (according to disease severity). All patients will receive standard anti-tuberculosis chemotherapy. Randomisation will be stratified by participating hospital/site, LTA4H genotype (CC or CT), and modified MRC disease severity grade ( Supplementary File 1) assessed on day 0 when consent is given.

The randomisation list will be computer-generated based on random permuted blocks with variable block size following Oxford University Clinical Research Unit (OUCRU) standard operating procedures. The OUCRU biostatistician in charge of randomisation list preparation will set up statistical code to generate the randomisation list and transfer it to the Study Pharmacist. The Study Pharmacist will then change the random seed, i.e. the initialisation of the random numbers generator, in the statistical code in order to blind the biostatistician and then run the code to prepare the final randomisation list. The generated randomisation lists will be securely incorporated within the trial database. A reliable manual back-up system will also be available. Randomisation to the three strategies for DILI will be 1:1:1 with stratification by initial randomisation (dexamethasone or placebo).

### Blinding, unblinding and treatment discontinuation

#### Blinding

All participants and investigators will be blinded to the treatment allocation. OUCRU clinical trials unit (CTU) pharmacists will create blinded drug packages (in fully made-up and labelled treatment packs) containing either active drugs or identical placebo sufficient for 6–8 weeks of treatment (dependent on the MRC grade of the participant) according to the prespecified randomisation list, and pre-ship them to the sites. After randomisation, the ward or trial research nurse will take the completed prescription form to the site pharmacy; they will dispense the trial-number specific pack containing the study drug. Unused drug will be returned to the site pharmacy if a participant withdraws from treatment. In ancillary study 3 MRI brain images will be read by an independent neuroradiologist, blinded to the treatment allocation and outcomes of the participant.

#### Unblinding

If, in the opinion of the local clinician, it is important for good clinical care to unblind treatment the documented request will be discussed with the site Principal Investigator (PI) and Chief Investigator (CI). If it is agreed that knowledge of treatment allocation is essential for the best management of the patient, the unblinding code will be provided by the study pharmacist holding the randomisation list at OUCRU CTU upon documented request from the CI. Generalised clinical deterioration is not sufficient for unblinding, given equipoise about the evidence base supporting the use of dexamethasone regardless of clinical severity. All instances of unblinding will be recorded and reported to the Data Monitoring Committee (DMC) and Trial Steering Committee (TSC).

#### Protocol treatment discontinuation

An individual participant may stop study drug early for any of the following reasons: Participant no longer believed to have TBM and all anti-tuberculosis treatment stopped; Unacceptable toxicity or adverse event; Intercurrent illness that prevents further treatment; Any change in the participant’s condition that justifies the discontinuation of treatment in the treating physicians opinion and after discussion with the site PI; Inadequate compliance with the protocol treatment in the judgement of the treating physician; Withdrawal of consent for treatment by the participant.

### Trial management

#### Interventions

All participants will receive the standard of care anti-tuberculosis drugs according to national guidelines. Each participant will be LTA4H genotyped. The enrolment and randomisation of the participant into the trial will be finalised according to genotype. TT-genotype participants will be treated with anti-tuberculosis drugs and open label dexamethasone ( Table 2). LTA4H CC- and CT-genotype participants will be randomised to receive dexamethasone or placebo for 6–8 weeks, in addition to anti-tuberculosis drugs, dependent upon disease severity ( Table 2). Study drug will be dispensed at randomisation from the site pharmacy in intravenous and oral (tablet) formulations. Placebo will be identical in appearance to active drug and dosed and dispensed in the same way.

**Table 2.** Study drug treatment regimen following randomisation.

	MRC Grade I Daily dexamethasone dose/route	MRC Grades II and III Daily dexamethasone dose/route
Week 1	0.3 mg/kg/24 hrs IV	0.4 mg/kg/24 hrs IV
Week 2	0.2 mg/kg/24 hrs IV	0.3 mg/kg/24 hrs IV
Week 3	0.1 mg/kg/24 hrs IV	0.2 mg/kg/24 hrs IV
Week 4	3mg/24 hrs oral	0.1 mg/kg/24 hrs IV
Week 5	2mg/24 hrs oral	4 mg/24 hrs oral
Week 6	1 mg/24 hrs oral	3 mg/24 hrs oral
Week 7	Stop	2 mg/24 hrs oral
Week 8		1 mg/24 hrs oral

Participants for the DILI strategy study will be randomised to one of three strategies ( Figure 2): Strategies are as follows:

1) Observe: measure transaminases, bilirubin, and INR every 3 days; do not change/stop anti-tuberculosis drugs unless transaminases rise to ≥10x normal, or total bilirubin rises >2.5mg/dl (>43 µmol/L), or INR >1.5 or symptoms of hepatitis worsen (nausea, vomiting, abdominal pain), in which case go to Strategy 3.
2) Stop pyrazinamide alone. Observe, measuring transaminases, bilirubin, and INR every 3 days. If transaminases do not fall to <5x ULN by day 5, or total bilirubin rises >2.5mg/dl (>43 µmol/L), or INR >1.5 or symptoms of hepatitis worsen at any time (nausea, vomiting, abdominal pain), go to Strategy 3.
3) Current standard of care (current US CDC guidelines ^9^, Supplementary File 2).

#### Anti-tuberculosis treatment

First line anti-tuberculosis treatment will follow current Vietnamese national guidelines. Rifampicin (10mg/kg/24 hrs; maximum 600mg), isoniazid (5mg/kg/24hrs; maximum 300mg), pyrazinamide (25mg/kg/24hrs; maximum 2g) and ethambutol (20mg/kg/24 hrs; maximum 1.2g) will be given for at least the first 2 months of treatment, provided drug resistance is not suspected or proven. Pyrazinamide will then be stopped and rifampicin, isoniazid and ethambutol (at the same doses) will then be given until at least 12 months anti-tuberculosis treatment in total has been given. The best treatment of TBM caused by isoniazid-resistant tuberculosis is uncertain, and the attending physician should decide which option to take. For participants with MDR tuberculosis, second-line treatment should be given as soon as possible, following national guidelines and local policies.

Participants with abnormal liver function tests (LFTs) at screening are eligible to enter the trial. These patients should be given standard first-line anti-tuberculosis treatment unless blood transaminase concentrations are ≥3 times the ULN with symptoms and signs of hepatitis (vomiting, abdominal pain, jaundice), or ≥5 times the ULN or a rise in serum bilirubin >2.0mg/dL (>34 µmol/L) without symptoms. In these participants, initial anti-tuberculosis treatment should consist of levofloxacin, ethambutol, and an aminoglycoside (either kanamycin, amikacin or streptomycin). LFTs should be monitored every 3 days and rifampicin started as soon as blood transaminases are <5x the ULN and/or the symptoms and signs of hepatitis resolve. Once the participant is tolerating a rifampicin-containing regimen isoniazid can be introduced and, if isoniazid is tolerated, the aminoglycoside can be stopped. If pyrazinamide is not used in treatment, at least 12 months of anti-tuberculosis treatment must be given. Participants with liver dysfunction at the start of treatment that require modified initial anti-tuberculosis treatment regimens are ineligible for the DILI strategy study.

#### Management of hepatitis B

Treating participants infected with hepatitis B (HBsAg positive) with corticosteroids carries a very small risk of reactivation and flare of hepatitis B infection ^10, 11^. In order to ensure the safety of patients we will:

1) Test all enrolled participants for HBsAg at baseline (day 0).
2) After randomisation (of CT or CC- genotypes) to dexamethasone or placebo, all HBsAg positive participants will be unblinded with respect to the study drug.
3) HBsAg positive participants randomised to dexamethasone (CT- or CC- genotypes) will receive tenofovir therapy at 300mg once daily. Tenofovir therapy will be for the duration of dexamethasone therapy, and then for a further 12 months after completion of dexamethasone therapy.
4) HBsAg positive participants (of any LTA4H genotype) randomised to placebo will NOT receive tenofovir therapy. Unblinding of HBsAg positive participants is necessary to identify those requiring tenofovir.
5) HbSAg positive TT- genotype participants, all of whom will receive open label dexamethasone, will also receive tenofovir therapy at 300mg once daily, according to the current standard of care for HBsAg positive patients as issued by the Vietnam Ministry of Health. Tenofovir therapy will be for the duration of dexamethasone therapy, and then for a further 12 months after completion of dexamethasone therapy.
6) LFTs and hepatitis B viral load testing will be performed in those participants with hepatitis B who are receiving dexamethasone. LFTs will be performed at least weekly until discharge, and then every 3 months for the full duration of the study. Liver function tests will be performed more frequently if there is a clinical need. Hepatitis B viral load testing will be performed every 3–6 months (following national guidelines) for the full duration of the study. Participants who are HBsAg negative, but HBcAb positive (i.e. evidence of past hepatitis B infection) will not receive tenofovir and will therefore not be unblinded, but they will be followed carefully for evidence of episodes of hepatitis. The monitoring of HBcAb positive participants (LFTs and hepatitis B viral load testing) will be as for HBsAg patients.

### Management of hepatitis C

Participants with hepatitis C infection (positive hepatitis C antibodies) will not be unblinded. However, they will be carefully monitored with liver function tests performed at least weekly until discharge, and then every 3–6 months for the full duration of the study. Liver function tests will be performed more frequently if there is a clinical need.

### Use of concomitant medication

All other concomitant medications essential for participant management are permitted at enrolment, subject to the exclusion criteria of no contraindications to the use of dexamethasone in the judgement of the attending clinician. If use of a concomitant medication that cannot safely be used with dexamethasone becomes essential after randomisation, then the study drug should be stopped and the concomitant medication used without unblinding. Drugs which increase the risk of gastrointestinal bleeding, such as non-steroidal anti-inflammatory drugs (NSAIDS), should be used with caution. Any other oral or intravenously administered corticosteroids are not permitted, unless deemed essential, in which case the study drug should be stopped and replaced by the chosen corticosteroid. Normal standards of clinical care should be followed.

### Management of neurological complications occurring after the start of anti-tuberculosis treatment

Corticosteroid use is not routine for the treatment of hydrocephalus or stroke, however for neurological deterioration secondary to tuberculomas most physicians recommend using corticosteroids. In this situation open-label dexamethasone is recommended. High-dose intravenous dexamethasone (0.4mg/kg/24hrs) should be prescribed, and the study drug stopped (if the study drug course is still ongoing).

## Data collection

The trial assessment schedule for the main trial is outlined in Table 3.

**Table 3.** Trial assessment schedule.

	DAYS					MONTHS				
	screening	0	R andom- isation	3	7	W eekly until discharge	30	60	M onthly to anti- TB drug end	12
ALL PARTICIPANTS										
Eligibility assessment	(X)									
Participant information sheet and consent	X									
Informed consent		X								
LTA4H genotype (5mls blood)		X								
Clinical assessment	(X)	X	X	X	X	X	X	X	X	X
Disability assessment							X	X	X	X
Chest X-ray	(X)							X		
Lumbar puncture (with paired plasma glucose)	(X)						X	X		
HIV test	(X)									
EDTA blood for genetic tests Full blood count Storage for later DNA extraction	(X)	X X			(X)					
Sodium Urea/Creatinine ALT/ bilirubin Hepatitis C antibodies Hepatitis B surface antigen Fasting blood sugar/HbA1c C-peptide/Lipids Strongyloides serology Serum Storage	(X) (X) (X)	X X X X X X			(X)	(X) (X) (X)		X X	(X) (X)	
Stool for Ova, cysts and parasites (Strongyloides) microscopy		X					X			
SUBSET OF PARTICIPANTS RECRUITED TO IMAGING STUDY (HTD only)										
Brain MRI		X						X		X
SUBSET OF PARTICIPANTS RECRUITED TO HYPONATRAEMIA/ICP SUB-STUDY (HTD only)										
24-hour fluid balance	(X)	X		X	X	X				
Plasma sodium Plasma osmolality		X		X	X	X				
Urinary sodium Urinary osmolality		X		X	X	X				
Plasma cortisol		X								
Doppler Ultra-sound assessment of intravascular volume		X		X	X	X				
Ultra-sound measurement of optic nerve sheath diameter		X		X	X	X				
SUBSET OF PARTICIPANTS RECRUITED TO ADRENAL SUPPRESSION SUB-STUDY (HTD only)										
Synacthen test						X (day 21)		X		

### Clinical assessment

Clinical assessment will include conscious level by GCS, new or ongoing focal neurological deficit, clinical treatment response, all serious adverse events, all adverse events of any grade leading to modification of anti-tuberculosis treatment or its interruption/early discontinuation (and clinician-assessed likelihood of relationship of adverse event to dexamethasone), and adherence to drugs (study drug and anti-tuberculosis drugs). Assessment of disability by the modified Rankin scale will be performed at day 30 from randomisation, monthly until completion of anti-TB drugs, and at month 12.

### Inpatient assessment

GCS and new focal neurology will be recorded daily during the participant’s hospital admission. Participants will be visited by one of the research team at screening, baseline, randomisation (when LTA4H genotype returns), and at least every 3 days for the first 4 weeks of treatment (unless they are discharged or die before 4 weeks) and then at least every 7 days whilst they remain in hospital. Formal trial clinical assessments will occur on day 0, 3, 7, 10, 14, and weekly thereafter until discharge (+/- 1 day).

### Outpatient assessment

After discharge clinical assessments will occur monthly until 12 months. Some of these assessments can be made by phone. Formal outpatient review will occur monthly (+/- 7 days) for at least the first 2 months following hospital discharge. The patient should have formal outpatient review at least every 2 months until month 12 after randomisation. The endpoints of survival and neurological disability by 12 months (-0/+1 month) should be assessed by formal outpatient review whenever possible.

### Liver function

Alanine transaminase (ALT) and bilirubin will be measured to evaluate liver toxicity every 7 days until discharge and at each subsequent follow-up visit until anti-tuberculosis treatment stops.

### Additional blood tests

EDTA blood will be taken for HBsAg, HBcAb, hepatitis B viral load, and hepatitis C antibodies. Serum will be stored for later DNA extraction when consent has been given.

### Glycosylated haemoglobin (HbA1c) and fasting blood sugar

HbA1c and fasting blood sugar will be measured at baseline and at 60 days from randomisation. To enable more detailed phenotyping of diabetes we will also measure C-peptide and blood lipids at baseline and store serum for future diabetes related auto-antibody testing.

### Strongyloides

All enrolled participants will be tested for serological evidence of Strongyloides infection at baseline (past or latent infection), and with stool examination for evidence of active infection at baseline and at 21–30 days from randomisation, depending on discharge date. When infection is detected, treatment with ivermectin will be provided. Stool will be examined for Strongyloides larvae at the end of study drug to determine whether reactivation alters TBM treatment responses.

### Synacthen test 

We will compare adrenal responsiveness at 3 weeks after randomisation and at the end of study drug treatment (6 or 8 weeks depending upon disease severity) in 100 consecutive patients using the short Synacthen test. The patient’s background cortisol level is measured by drawing 2mls of blood at 0900hrs. 250mcg of Synacthen is then administered intravenously; 3ml samples of blood are taken at thirty minutes and sixty minutes to measure the cortisol level after this adrenal stimulation. The Synacthen test will be repeated at day 60 after randomisation.

### Lumbar puncture

Lumbar puncture should be performed, unless clinically contraindicated, as part of routine clinical care for the baseline assessment, and on days 30 and 60 after randomisation to assess treatment response. Opening pressure should be measured, at least 5mls of CSF should be taken for mycobacterial investigations alone, and assessments of cell count and differential, protein, glucose, and lactate should also be performed on 1–2 mls of additional CSF. Ziehl Neelsen stain, GeneXpert, and *M. tuberculosis* culture will be performed on all CSF taken. As part of an ancillary study of the impact of dexamethasone on CSF inflammation and gross cerebral pathology we will measure concentrations of a variety of inflammatory mediators in the CSF (leucocytes, cytokines, chemokines, and eicosanoids, for example) at baseline and on days 30 and 60 after randomisation to determine how dexamethasone influences their expression.

### Hyponatraemia and raised intracranial pressure

The rapid diagnosis of intracranial hypertension is challenging and urgently required in TBM. In the subset of participants enrolled to HTD, Ho Chi Minh City, Vietnam, we will investigate the pathophysiology of TBM-associated hyponatraemia by serial assessments of fluid balance, paired plasma and urinary sodium and osmolality, and intravascular volume by Doppler ultrasound assessment of inferior vena cava collapsibility index ^12, 13^. We will also use portable ultrasound to measure the optic nerve sheath diameter, which has been shown to be a reliable and non-invasive measure of raised intracranial pressure ^14, 15^. These additional measurements will be assessed at days 0, 3, and 7, and weekly until discharge. Plasma cortisol will be measured at day 0.

### Imaging

Chest Xray will be performed at screening and on day 60 after randomisation. Brain imaging by MRI (or CT if the participant cannot tolerate an MRI) will be performed at baseline (+/- 7 days), 60 days (+/- 7 days), and at 12 months (-0/+1 month). We will investigate whether dexamethasone influences the incidence and outcome of hydrocephalus, infarcts and tuberculoma formation in these participants.

## Adverse events and safety reporting

### Adverse events

Specific procedures will be followed when notifying and reporting adverse events (AEs) or adverse reactions (ARs). The definitions of the EU Directive 2001/20/EC Article 2 based on the principles of ICH good clinical practice (GCP) apply to this trial protocol. All AEs and ARs will be assessed as to whether they are serious or not. If the event is serious and not only related to TBM, or is fatal, then a serious adverse event (SAE) form must be completed and the OUCRU CTU notified within 24 hours. All AEs and ARs (serious and non-serious) should be graded using toxicity gradings.

Causality of all SAEs or serious adverse reactions (SARs) in relation to the trial therapy (dexamethasone) will be assessed. There are five categories: unrelated, unlikely, possible, probable, and definitely, related. If the causality assessment is unrelated or unlikely to be related, the event is classified as an SAE. If the causality is assessed as possible, probable or definitely related, then the event is classified as an SAR. If there is at least a possible involvement of the trial treatment (or comparator), the investigator must assess the expectedness of the event. An unexpected adverse reaction is one not previously reported in the current Summary of Product Characteristics (SPC) at the time the event occurred, or one that is more frequent or more severe than previously reported. If a SAR is assessed as being unexpected, it becomes a suspected unexpected serious adverse reaction (SUSAR). Investigators should always check the current version of the SPC.

### Safety reporting

The OUCRU CTU is responsible for the reporting of SUSARs and other SARs to the regulatory authorities and the research ethics committees. The following events will be reported to the relevant authorities in Vietnam: All unexpected SAEs, all SAEs judged to be related or possibly related to the trial intervention, and all deaths. All SAEs will be reported to OxTREC (Oxford Tropical Research Ethics Committee) in the annual review form and to the DMC in accordance to the DMC charter. An independent DMC will oversee the safety of the trial.

### Interim analyses

Interim analyses are planned 6-monthly during the first two years of recruitment and yearly thereafter until the completion of the trial but the DMC has the authority to modify the frequency of interim analyses. At these interim analyses, the DMC will receive a report including unblinded summaries of baseline characteristics, the primary endpoint, overall survival, and adverse events by treatment arm as well as conditional power curves for the primary endpoint. Statistical summaries will be provided both for all randomised subjects and for the subgroup of participants with CC and CT-genotype. The DMC may recommend termination or modification of the study in the circumstances that dexamethasone is harmful (placebo superior), or that dexamethasone is beneficial. The Haybittle-Peto boundary, requiring p < 0.001 at interim analysis to consider stopping for efficacy, should be used as guidance in both directions. This boundary may apply to the overall population with CC or CT genotype combined or either genotype alone. Importantly, a DMC recommendation will not be based purely on statistical tables, but will also require clinical judgment. The DMC will also review data from those enrolled into the drug-induced liver injury sub-study, in particular the incidence of acute hepatic failure in each of the management strategy arms.

## Statistical analysis

### Sample size justification – main trial

In a previous TBM trial ^4^, 73/189 (38.6%) of HIV-negative subjects with CC-genotype and 69/214 (32.2%) with CT-genotype experienced a neurological event or died during the 9 month follow-up period. Only few additional events are expected between months 9 and 12 of follow-up. All subjects in this trial received dexamethasone. In principle, administration of dexamethasone in CC and CT- genotypes would be discouraged if placebo could be shown to be non-inferior to dexamethasone. However, as the benefit of dexamethasone in the TT-genotype is undisputed and personalised administration of dexamethasone in HIV-uninfected subjects would necessitate rapid genotype testing, some evidence of harm of dexamethasone in the CC/CT population (or the CC group alone) is required. Therefore we have opted for a hybrid trial-design approach which assumes a modest harm of dexamethasone and aims to prove non-inferiority of placebo first but also allows claiming superiority of placebo in case dexamethasone proves to induce substantial harm ^16^. Moreover, as it is possible that harm of dexamethasone only applies to the CC-genotype, the trial should allow dropping the CT group at an interim analysis but continue randomisation of the CC group. To protect the one-sided overall familywise error rate of 2.5% for the analysis of the primary endpoint across the two co-primary populations (the full CC/CT population and the CC population), we will assign a multiplicity-corrected one-sided significance level of 2% to the full population and 0.87% to the CC population exploiting the correlation between test statistics on the two populations using the Spiessens and Debois method ^17^.

We set the non-inferiority margin in favour of dexamethasone at a hazard ratio (HR) of 0.75 and assume a true HR of 1.15 in the CC/CT population. Assuming an absolute risk of a neurological event or death in the dexamethasone group by 12 months of 35%, a HR=1.15 corresponds to risk of 31.2% on placebo, and the non-inferiority margin implies that we can exclude an absolute risk increase of placebo of (at worst) +8.7%. Under these assumptions a total of 184 neurological events or deaths would be required to obtain 80% power at the one-sided 2% significance level. Assuming an overall event risk of ≥32%, and an 11% sample size increase to compensate for loss-to-follow-up and reductions in power due to the allowance for stopping due to futility, a total of 640 HIV-negative subjects with CC or CT-genotype will be randomised into the trial. Based on experience from our earlier TBM trials ^4, 7, 18, 19^, only a very low number of subjects will be excluded from the per-protocol population, hence only minimally reducing power in this population. Of note, with 184 neurological events or deaths (half of them in the CC group), the trial also has 80% power to prove harm of dexamethasone in the CC/CT group or the CC group in case the true HR is at least 1.54 in all subjects or 1.96 in CC patients, respectively. Our recent trial enrolled 468 HIV-uninfected adults with TBM in 30 months and 90% of them had CC or CT-genotype. Assuming similar recruitment rates, enrolment will be complete within 4 years.

### Sample size justification – DILI strategy study

A review of 36 subjects who interrupted rifampicin and isoniazid because of clinical hepatitis or jaundice events from our previous trial ^4^ gave the following data: the median (interquartile range (IQR)) onset date of the drug-related liver injury was 50 (15–84) days from initiation of anti-tuberculosis treatment, the median (IQR) duration of the rifampicin and isoniazid interruption was 16 (12–24) days, and 12 subjects subsequently died (8 of them within 60 days). Of note, the duration of the treatment interruption was <t;30 days for 32 (89%) of the 36 subjects and the remaining 4 subjects never re-started rifampicin or isoniazid but continued to receive alternative anti-tuberculosis treatment for >100 days.

We hypothesise that strategies 1 and 2, respectively, will result in a relative reduction in the duration of the treatment interruption of 50% for subjects with interruptions <30 days, but that they do not affect longer interruptions (as the corresponding subject might have permanent intolerance to rifampicin and isoniazid) or mortality. Based on simulations of hypothetical trials using re-sampling from the data described above, the hypothesised treatment effect, and the Wilcoxon rank sum test for analysis, we determined that the power to detect such an effect size with a sample size of at least 50 subjects per arm is >85%. Of note, given this is an ancillary and essentially ‘opportunistic’ study, we have chosen a liberal (i.e. not multiplicity corrected) two-sided significance level of 5% for each of the two primary comparisons of strategies 1 and 2, respectively, versus strategy 3.

### Analysis populations – main trial

As the effect of dexamethasone may depend on the genotype with worse anticipated outcomes for CC-genotype, this trial has two co-primary analysis populations: The full analysis population containing all randomised patients and the subgroup of subjects with CC-genotype. Analyses in the subgroup with CT-genotype will also be performed but are exploratory only. In addition, the primary end point will be analysed in the per-protocol population, which will exclude the following patients: patients with a final diagnosis other than TBM, major protocol violations and those receiving less than 1 week of administration of the randomised study drug for reasons other than death. In all populations, patients will be analysed according to their randomised arm. For the analysis of the primary endpoint, a multiplicity corrected one-sided significance level of 2% for the full population and 0.87% for the CC subgroup will be applied as described in the sample size justification above. For all other analyses, the conventional two-sided significance level of 5% will be applied. Published diagnostic criteria ^20^ will be applied to all enrolled participants at the end of the study when all mycobacterial culture results are available ( Supplementary File 3). The criteria will sub-divide all cases into definite, probable and possible TBM, and those with an alternative diagnosis.

For the primary analyses of the main trial the second randomisation in the DILI strategy study will be ignored and the estimated dexamethasone treatment effect can thus be interpreted as an average effect across the three management strategies. We believe that this is justified because only approximately 70 (11%) subjects are expected to be enrolled in the nested trial with roughly similar numbers from both arms, because the efficacy of the different management strategies is unlikely to depend on whether the patient received dexamethasone or not as it tests a very different intervention, and because the anticipated effect of the management strategy on neurological events and deaths is relatively small. However, in a supplementary analysis, we will also compare the primary endpoint between the treatment policies “dexamethasone treatment plus standard of care management of drug-related liver injury” vs. “placebo treatment plus standard of care management of drug-related liver injury” using an inverse probability weighting based analytical framework ^21^.

### Analysis populations – DILI strategy study

All patients in the DILI strategy study will be analysed according to their randomised arm as an intention-to-treat (ITT) analysis. The two primary comparisons are the comparisons of strategies 1 and 2, respectively, versus strategy 3 (with tests conducted at the unadjusted two-sided 5% significance level) and comparisons between strategies 1 and 2 will be exploratory only.

### Primary endpoint analysis – main trial

The primary endpoint of this trial is the time to death or a new neurological event during 12 months of follow-up. The primary endpoint will be analysed using a Cox proportional hazards regression model with treatment as the only covariate and stratification by TBM MRC severity grade at enrolment (I, II, or III) and LTA4H genotype (CC or CT). The primary effect measure is the resulting hazard ratio comparing dexamethasone vs. placebo with a corresponding confidence interval and p-value. Non-inferiority of placebo in the entire population or the CC-genotype subgroup will be established if the corresponding test (at the one-sided 2% or 0.87% significance level for the full population and the CC subgroup, respectively) rejects the null hypothesis that dexamethasone decreases the hazard of the primary endpoint by 25% or more. Superiority of placebo will additionally be established, if the null hypothesis that dexamethasone does not affect the hazard of the primary endpoint can be rejected against the one-sided alternative that dexamethasone causes harm. The distribution of the primary endpoint will also be visualized using Kaplan-Meier plots and explicit survival estimates at 3, 6, 9, and 12 months of follow-up will also be calculated.

The proportional hazards assumption will be formally tested based on scaled Schoenfeld residuals and visually assessed by a plot of the scaled Schoenfeld residuals versus transformed time. In case of a significant test, a formal comparison of the absolute risk of an event by 12 months between the two groups will also be performed (using a Wald-type test based on Kaplan-Meier estimates at 12 months and associated standard errors using Greenwood’s formula). The homogeneity of the treatment effect on the primary endpoint across subgroups will be assessed by subgroup analyses and formal tests of interactions between treatment and the following grouping variables: genotype (CC or CT), TBM MRC severity grade at enrolment (I, II, or III), and drug resistance pattern (MDR-TB or rifampicin mono-resistance, isoniazid resistant non-MDR, no or other resistance). To obtain an adjusted treatment effect estimate and to assess the effect of other covariates, the primary endpoint will also be modelled using a multivariable Cox proportional hazards regression model including the following covariates (in addition to the treatment group): genotype, TBM MRC severity grade at enrolment, and drug resistance pattern.

### Primary endpoint analysis – DILI strategy study

For the analysis of the primary endpoint of the DILI strategy study, the non-parametric Wilcoxon rank sum test will be used for pairwise comparisons. An additional adjusted analysis (with adjustment for the initial randomisation, HIV-status, and the time from initial randomisation to the second randomisation) will be also be performed treating the outcome as an ordinal outcome and using a proportional odds logistic regression model (which can be interpreted as an extension of the Wilcoxon rank sum test).

### Secondary endpoint analysis

Overall survival will be analysed in the same way as the primary endpoint (except that non-inferiority will not be formally assessed). Neurological disability (as assessed by the ordinal modified Rankin scale) at 12 months will be compared between the two arms with a proportional odds logistic regression model with the treatment assignment as the main covariate and adjustment for genotype and TBM MRC severity grade. The result will be summarised as a cumulative odds ratio with a corresponding 95% confidence interval and p‐value. Patients with a missing 12 month disability assessment will be excluded from the main analysis but an alternative analysis based on multiple imputation (including disability assessments at earlier time points in the imputation model) will also be performed. The number and proportion of subjects requiring ‘rescue’ corticosteroids will be summarised by treatment arm. Comparisons will be based on logistic regression with the treatment assignment as the main covariate and adjustment for genotype and TBM MRC severity grade.

### Analysis of adverse events

The number of patients with any adverse events and specific events, respectively, will be summarised and informally compared between the two treatment arms based on Fisher’s exact test. The total number of adverse event episodes per patient will also be summarised and informally compared based on a quasi-Poisson regression model with treatment as the only covariate. The following subgroups of adverse events will also be separately summarised: grade 3&4 adverse events; serious adverse events; serious adverse events possibly, probably, or definitely related to the study drug; adverse events leading to TB treatment interruptions. Grade 3&4 laboratory abnormalities will be summarised in the same way as clinical adverse events.

### Baseline descriptive analyses

Baseline characteristics will be summarised as median (lower and upper quartiles) for continuous data and frequency (percentage) for categorical data. The amount of missing data for each baseline characteristic will also be displayed.

## Ethical considerations

### Confidentiality

Participants’ confidentiality will be maintained throughout the trial. Participants will be assigned a trial identification number and this will be used on case report forms (CRFs); participants will not be identified by their name. The investigator will securely keep a participant trial register showing identification numbers, surnames and date of birth. The unique trial number will identify all laboratory specimens, case record forms, and other records and no names will be used, in order to maintain confidentiality. Data submitted to OUCRU CTU and samples sent to central testing facilities will be identified only by the trial number and participant initials.

### Consent

Written informed consent must be obtained in order to enter into the trial and be randomised. If a participant lacks capacity, written consent must be obtained from a person with responsibility (e.g. family member/relative), in their own language before enrolment by the site PI or an appropriately trained doctor. All potential participants (or their families) will be given a participant information sheet clearly listing the risks and benefits of the trial. All potential participants (or their families) will be able to discuss participation with their consulting doctor who will be able to address questions not covered or arising from the participant information sheet. Incapacitated adults with TBM will be eligible to enter the trial. These adults have more severe disease and therefore may benefit most from adjunctive dexamethasone. We anticipate around 70% of participants with TBM will lack capacity at the start of treatment. An option will be given to patients to enrol in the main study, but not the ancillary studies.

If consent is provided by a relative, the participant should be consulted and consent recorded if and when they have the capacity to do so. If they are happy to remain in the trial, the participant should complete a participant consent form at this time. If they wish to withdraw from the trial, no further trial-related procedures will be performed, but data to this point would be used in analysis. Data from any participant who dies before regaining capacity (but whose family member has provided consent) will be included in analysis.

### Ethical approval

The trial protocol has been approved by the Oxford Tropical Research Ethics Committee, the Ethics Committees of the Hospital for Tropical Diseases Pham Ngoc Thach Hospital and the Vietnam Ministry of Health.

### Protocol violations

All deviations from protocol will be addressed in source documents and reported to the OUCRU CTU.

### Withdrawing from the trial

A participant (or their relative) is free to refuse to participate in or withdraw from all or any aspect of the trial, at any time and for any reason. If a participant chooses to discontinue their trial treatment they should always be followed up (providing they are willing) and they should be encouraged not to leave the whole trial. If they do not wish to remain on trial follow-up however, their decision must be respected and the participant will be withdrawn from the trial. Participants may change their minds about stopping trial follow-up at any time and re-consent to participation in the trial.

### Data collection and storage

Clinical data and clinical laboratory data will be entered into CliRes, a 21 CFR Part 11-compliant data capture system provided by the OUCRU information technology department. The data system includes password protection and internal quality checks, such as automatic range checks, to identify data that appear inconsistent, incomplete, or inaccurate. Trial data will be recorded onto paper CRFs and entered into CliRes. The participants will be identified by a unique trial specific number and/or code in any database. The name and any other identifying detail will not be included in any trial data electronic file. CRFs, clinical notes and administrative documentation will be kept in a secure location and held for 15 years after the end of the trial. Clinical information will not be released without written permission, except as necessary for monitoring, auditing and inspection purposes. Electronic data will be kept for at least 20 years at the OUCRU CTU.

### SPIRIT checklist

A SPIRIT checklist for this trial protocol is attached ( Supplementary File 4).

### Trial Committees

A Trial Management Group (TMG) will be formed to conduct the day-to-day management of the trial at the OUCRU CTU. This will include the CI, Head of OUCRU CTU, Trial Statistician, Clinical Project Manager, Trial Manager and Data Manager. The group will meet at least once per month, although may meet more or less often as required. The TSC has membership from the TMG plus independent members (Professor Nicholas Paton (Infectious Diseases physician and Clinical Trialist, National University of Singapore, Singapore), Professor Ben Marais (Senior Tuberculosis Researcher and Trialist, University of Sydney, Australia), DrTruong Huu Khanh (Infectious Diseases Physician, Paediatric Hospital Number 1, Ho Chi Minh City, Vietnam)), including the Chair (Professor Robert Wilkinson (Honorary Professor and Director Wellcome Centre for Infectious Diseases Research in Africa, University of Cape Town, South Africa)).The role of the TSC is to provide overall supervision for the trial and provide advice through its independent chair. The ultimate decision for the continuation of the trial lies with the TSC. The DMC (Professor Sarah Walker (DMC Chair, Senior Statistician and Clinical Trialist, MRC Clinical Trials Unit, University College London), Professor Graeme Meintjes (Senior Infectious Diseases/HIV Physician, University of Cape Town, South Africa) and Professor Nina Ruslami (Senior TBM Clinician and Researcher, Universitas Padjadjaran, Bandung, Indonesia)), will advise the TSC and can recommend premature closure or reporting of the trial, or that recruitment be discontinued or modified. The DMC will advise the TSC and can recommend premature closure or reporting of the trial, or that recruitment be discontinued or modified. The DMC is independent from the sponsor. Access to interim data and results will be confidential and strictly limited to the DMC and results (except for the recommendation) will not be communicated to the outside and/or clinical investigators involved in the trial. This trial is sponsored by The University of Oxford (Contact: University of Oxford, Research Services, University Offices, Wellington Square, Oxford OX1 2JD, Tel +44 (0) 1865 282585).

## Data dissemination

Manuscripts arising from the trial will, wherever possible, be submitted to peer-reviewed journals which enable Open Access via UK PubMed Central within six months of the official date of final publication. In line with research transparency and greater access to data from trials OUCRU’s clinical trials are registered at ClinicalTrials.gov and a data sharing policy is in place. Data exchange complies with Information Governance and Data Security Policies in all of the relevant countries.

## Discussion

Dexamethasone has been shown to improve survival in HIV-uninfected individuals with TBM ^12^. Previous data strongly suggests hyperinflammatory LTA4H TT-genotype patients with TBM benefit from dexamethasone, and that adjunctive dexamethasone does not benefit, and may cause harm, when given to patients with LTA4H CT or CC-genotype ^5^. How corticosteroids improve survival in TBM in HIV-uninfected patients, and whether they do so in all HIV-uninfected patients, remains uncertain and is the focus of the LAST ACT trial. Adjunctive dexamethasone is currently standard of care for HIV-unaffected patients with TBM. This study may identify a role for using LTA4H genotype to guide adjunctive anti-inflammatory therapy.

## Trial status

Trial protocol version 1.6. Estimated recruitment start date 1 ^st^ February 2018. Estimated time for recruitment is 4 years.

## Ethics statement

The trial has ethics approval from the Oxford Tropical Research Ethics Committee (approval number 52-16), the Ethics Committees of the Hospital for Tropical Diseases (approval number CS/ND/17/27) and Pham Ngoc Thach Hospital (approval number CS/PT/17/06), and the Vietnam Ministry of Health.

## Data availability

No data are associated with this article.

## Disclosures

Due to the linked nature of this trial, some sections of this protocol also form part of the linked RCT ACT HIV (Trial registration number: NCT03092817), which has also been submitted to *Wellcome Open Research* ^8^. ACT HIV is a parallel group, randomised (1:1), double blind, placebo-controlled multi-centre Phase III trial, comparing the effect of dexamethasone versus placebo on overall survival in HIV-infected patients with TBM. The 7 ancillary studies in LAST ACT are also recruited to through the ACT HIV trial. As such the hypotheses, design, methods, sample size justification; analysis plans and endpoints of these ancillary studies will also be described in the ACT HIV trial, and will appear identically here. LAST ACT follows the same OUCRU protocols and local / national guidelines as ACT HIV, therefore randomisation, blinding and unblinding procedures, adverse event and safety reporting, ethics and confidentiality sections also appear identically here for LAST ACT as for the ACT HIV trial.

## Supplementary material

Supplementary File 1. Modified MRC grade of TBM.

Supplementary File 2. Current standard of care in DILI.

Supplementary File 3. TBM diagnostic criteria.

Supplementary File 4. SPIRIT checklist.

Supplementary File 5. LAST ACT consent form for HTD Vietnam.

## References

[1] Thwaites GE: Advances in the diagnosis and treatment of tuberculous meningitis. *Curr Opin Neurol.* 2013;26(3):295–300. 10.1097/WCO.0b013e3283602814 23493162

[2] Prasad K Singh MB Ryan H: Corticosteroids for managing tuberculous meningitis. *Cochrane Database Syst Rev.* 2016;4:CD002244. 10.1002/14651858.CD002244.pub4 27121755 PMC4916936

[3] Thwaites GE van Toorn R Schoeman J: Tuberculous meningitis: more questions, still too few answers. *Lancet Neurol.* 2013;12(10):999–1010. 10.1016/S1474-4422(13)70168-6 23972913

[4] Heemskerk AD Bang ND Mai NT: Intensified Antituberculosis Therapy in Adults with Tuberculous Meningitis. *N Engl J Med.* 2016;374(2):124–34. 10.1056/NEJMoa1507062 26760084

[5] Tobin DM Roca FJ Oh SF: Host genotype-specific therapies can optimize the inflammatory response to mycobacterial infections. *Cell.* 2012;148(3):434–46. 10.1016/j.cell.2011.12.023 22304914 PMC3433720

[6] Tobin DM Vary JC Jr Ray JP: The *lta4h* locus modulates susceptibility to mycobacterial infection in zebrafish and humans. *Cell.* 2010;140(5):717–30. 10.1016/j.cell.2010.02.013 20211140 PMC2907082

[7] Thwaites GE Nguyen DB Nguyen HD: Dexamethasone for the treatment of tuberculous meningitis in adolescents and adults. *N Engl J Med.* 2004;351(17):1741–51. 10.1056/NEJMoa040573 15496623

[8] Donovan J: Adjunctive dexamethasone for the treatment of HIV-infected adults with tuberculous meningitis (ACT HIV): study protocol for a randomised controlled trial. [version 1; referees: awaiting peer review]. *Wellcome Open Res.* 2018;3:XX. 10.12688/wellcomeopenres.14006.1 PMC6143919 30320225

[9] Masur H Brooks JT Benson CA: Prevention and treatment of opportunistic infections in HIV-infected adults and adolescents: Updated Guidelines from the Centers for Disease Control and Prevention, National Institutes of Health, and HIV Medicine Association of the Infectious Diseases Society of America. *Clin Infect Dis.* 2014;58(9):1308–11. 10.1093/cid/ciu094 24585567 PMC3982842

[10] Kim TW Kim MN Kwon JW: Risk of hepatitis B virus reactivation in patients with asthma or chronic obstructive pulmonary disease treated with corticosteroids. *Respirology.* 2010;15(7):1092–7. 10.1111/j.1440-1843.2010.01798.x 20630033

[11] Lai CL Yuen MF: Systemic corticosteroid and reactivation of chronic hepatitis B. *Respirology.* 2010;15(7):1017–8. 10.1111/j.1440-1843.2010.01822.x 20874743

[12] Hutchings S Bisset L Cantillon L: Nurse-delivered focused echocardiography to determine intravascular volume status in a deployed maritime critical care unit. *J R Nav Med Serv.* 2015;101(2):124–8. 10.1186/2197-425X-3-S1-A919 26867411

[13] Marik PE: Techniques for assessment of intravascular volume in critically ill patients. *J Intensive Care Mede.* 2009;24(5):329–37. 10.1177/0885066609340640 19648183

[14] Komut E Kozaci N Sonmez BM: Bedside sonographic measurement of optic nerve sheath diameter as a predictor of intracranial pressure in ED. *Am J Emerg Med.* 2016;34(6):963–7. 10.1016/j.ajem.2016.02.012 26944107

[15] Aduayi OS Asaleye CM Adetiloye VA: Optic nerve sonography: A noninvasive means of detecting raised intracranial pressure in a resource-limited setting. *J Neurosci Rural Pract.* 2015;6(4):563–7. 10.4103/0976-3147.165347 26752428 PMC4692017

[16] Freidlin B Korn EL George SL: Randomized clinical trial design for assessing noninferiority when superiority is expected. *J Clin Oncol.* 2007;25(31):5019–23. 10.1200/JCO.2007.11.8711 17971602

[17] Spiessens B Debois M: Adjusted significance levels for subgroup analyses in clinical trials. *Contemp Clin Trials.* 2010;31(6):647–56. 10.1016/j.cct.2010.08.011 20832503

[18] Torok ME Yen NT Chau TT: Timing of initiation of antiretroviral therapy in human immunodeficiency virus (HIV)--associated tuberculous meningitis. *Clin Infect Dis.* 2011;52(11):1374–83. 10.1093/cid/cir230 21596680 PMC4340579

[19] Thwaites GE Bhavnani SM Chau TT: Randomized pharmacokinetic and pharmacodynamic comparison of fluoroquinolones for tuberculous meningitis. *Antimicrob Agents Chemother.* 2011;55(7):3244–53. 10.1128/AAC.00064-11 21502621 PMC3122453

[20] Marais S Thwaites G Schoeman JF: Tuberculous meningitis: a uniform case definition for use in clinical research. *Lancet Infect Dis.* 2010;10(11):803–12. 10.1016/S1473-3099(10)70138-9 20822958

[21] Lokhnygina Y Helterbrand JD: Cox regression methods for two-stage randomization designs. *Biometrics.* 2007;63(2):422–8. 10.1111/j.1541-0420.2007.00707.x 17425633

