# Supplementary material for: Adjunctive dexamethasone for the treatment of HIV-uninfected adults with tuberculous meningitis stratified by Leukotriene A4 hydrolase genotype (LAST ACT): Study protocol for a randomised double blind placebo controlled non-inferiority trial
Source: Wellcome Open Res. 2018 Mar 20;3:32. [Version 1] doi: 10.12688/wellcomeopenres.14007.1 (PMC6182672; doi:10.12688/wellcomeopenres.14007.1)
Supplement: Supplementary file 1 [file wellcomeopenres-3-15224-s0000.tgz › 9c69e5a7-fd87-4514-ae3f-aab919f05d47.docx]

**Modified British Medical Research Council (MRC) TBM severity grades**

Grade I. GCS 15; no focal neurological signs.

Grade II. GCS 11-14, or 15 with focal neurological signs.

Grade III. GCS≤10 with or without focal neurological signs.
