# Supplementary material for: Adjunctive dexamethasone for the treatment of HIV-uninfected adults with tuberculous meningitis stratified by Leukotriene A4 hydrolase genotype (LAST ACT): Study protocol for a randomised double blind placebo controlled non-inferiority trial
Source: Wellcome Open Res. 2018 Mar 20;3:32. [Version 1] doi: 10.12688/wellcomeopenres.14007.1 (PMC6182672; doi:10.12688/wellcomeopenres.14007.1)
Supplement: Supplementary file 5 [file wellcomeopenres-3-15224-s0004.tgz › 256361b3-db0d-4245-bb07-295c524db2e5.doc]

**PATIENT INFORMATION SHEET: A TRIAL OF STEROIDS FOR TB MENINGITIS**

**For patients enrolled in HTD, Vietnam only**

**Leukotriene A4 hydrolase Stratified Trial of Adjunctive Corticosteroids for HIV-uninfected adults with Tuberculous meningitis (The LAST ACT trial)**

You are being invited to take part in a research study of adults with tuberculous (TB) meningitis. Approximately 720 people will be a part of this study which is being conducted by the Oxford University Clinical Research Unit (OUCRU) in Vietnam. The study is being performed in two hospitals in Ho Chi Minh City: the Hospital for Tropical Diseases and Pham Ngoc Thach Hospital.

Please read this information sheet carefully or have someone read it to you. You will be given a copy of this form to keep.

**What is the reason for doing the study?**

TB-meningitis is an infection of the brain caused by tuberculosis. We want to improve the treatment of TB-meningitis. Normally, TB-meningitis patients will be given antibiotics for at least 9 months to kill the infection. However, we want to know whether we should add a drug – called dexamethasone – to help control the inflammation in the brain at the start of treatment whilst the antibiotics kill the infection.

We have conducted many previous studies of TB-meningitis in Vietnam. These studies have shown that some people appear to benefit from adding dexamethasone to TB drug treatment, but others do not. We have tried to work out why this happens and we have discovered that people have a gene (part of the genetic code that makes us who we are) which may determine whether dexamethasone helps in the treatment of TB-meningitis, or does not help. The gene (which is called ‘Leukotriene A4 hydrolase’) varies between people: in around 15% of Vietnamese people the gene seems to make dexamethasone very effective in TB-meningitis treatment, but in the rest it is very uncertain whether dexamethasone is effective and may even be harmful. Therefore, in this study we want to find out whether we can use this gene to predict whether dexamethasone will make you better from TB-meningitis.

There is another reason for doing the study. One of the problems with the standard antibiotics for tuberculosis treatment is that they can upset your liver. In about one person in ten, the liver can become inflamed by the drugs and the drugs may need to be stopped. If the drugs are stopped the infection in the brain can come back. We want to see which of three different options for dealing with the liver inflammation is best.

Lastly, we want to work out how dexamethasone might work to help patients with TB patients. With your consent, we would like to take X-ray images of your brain and we will use ultrasound to measure the pressure in your brain and the amount of fluid in your body. We also want to see what effect dexamethasone has on the hormones in your body. None of these interventions will cause you pain or discomfort, but they will allow us to monitor you very closely and to detect and treat any complications of the disease should they occur.

**What will happen if I take part in the study?**

First, we will take a small sample of your blood and test it for the gene in our laboratories in the city. We will get the results back from this test within 48 hours. If you are one of the 15% of the Vietnamese people who have the type of the gene which we believe makes dexamethasone effective then we will treat you with the best available TB drugs (for at least 12 months) and dexamethasone (for the first 6-8 weeks).

However, if you have one of the other forms of the gene we will treat you with best available TB drugs and ***either*** dexamethasone ***or*** a harmless ‘placebo’. You will have an equal chance of being treated with either dexamethasone or placebo but neither you nor your doctor will know which one you receive. The placebos are injections and pills that will look identical to the dexamethasone injections and pills, but they will not contain any drug (they are harmless).

At the end of the trial the investigators will find out which treatment you and everyone else in the trial received and they will be able to calculate whether dexamethasone helped patients get better, or not. This is the best way to study a treatment, because it gives the most accurate answer on which treatment is best.

We want to understand how dexamethasone might work; therefore we will follow your response to treatment very closely. As long as it is safe to do so, we will scan your brain at the start of the study and after 2 and 12 months. These scans will help us monitor your treatment carefully and allow us to detect and manage any complications quickly. They do not cause any pain or discomfort. If you would like to participate in the trial but do not agree to having brain images taken, you can decline consent by checking the checkbox on the consent form.

In addition, we will perform tests on your blood and urine to monitor the amount of salt in your body and to see how dexamethasone affects the hormones in your body. Patients with TB meningitis often lose salt and this can make them very unwell. We want to work out why this happens and what to do about it. We will use an ultrasound machine (which is harmless and does not cause any pain or discomfort) to see how much fluid you have in your body. We will also use the ultrasound machine to see if your brain pressure is high by looking at the back of your eyes. In addition, we will test how dexamethasone affects the hormones produced by your adrenal gland (a small gland just above the kidney which produces steroid hormones). We will do this by giving you a small injection of a substance that stimulates the adrenal gland to produce hormones. We will test your blood before and after the injection and see whether dexamethasone has changed the way the adrenal gland produces hormones. The test is completely safe and will be performed after 3 weeks and at the end of dexamethasone treatment.

If you would like to participate in the trial but do not agree to having the salt studies and ultrasounds performed, or the hormone study, you can decline consent by checking the checkbox on the consent form.

Regular care for TB meningitis includes blood tests and tests on the spinal fluid in your back. To take this fluid, doctors will put a needle in your back. Blood and spinal fluid samples taken for study purposes will be taken as the same time as routine tests. Therefore, you don’t get extra needle pokes for these tests. We will also check a stool sample to make sure that you do not have a common parasite infection that may complicate your TB meningitis treatment. If you have this infection, we will treat it in the normal way.

After you leave hospital, you will be given TB medicine to take every day and you will be followed up in the clinic every month. You may also be given study tablets to take for a few days or weeks. It is very important you take TB and study medicines as instructed. During your visits, some tests will be done to check your health.

The entire study lasts 12 months when treatment and testing will be finished. Your treatment for TB meningitis will last 9-12 months, regardless of whether you enter the study.

If you need treatment for any other conditions during the trial, this will be given in the normal way. Being in the trial will not affect how you are treated for any other health problem you might have.

If your liver gets upset by the tuberculosis antibiotics at any time during treatment we may need to stop the drugs. We are not sure whether to stop all of them immediately, or wait and just stop one or two. Therefore, with your consent, if your liver does get upset one of these three different drug-stopping options will be selected for you. Like the dexamethasone treatment, which option you receive will be by chance (random). We believe all three options are safe, but some of the options may allow you to have the best antibiotics for longer whilst still allowing your liver to recover. If you would like to participate in the trial but do not agree to taking part in this part of the trial, you can decline consent by checking the checkbox on the consent form.

**What tests will be done and what are their risks?**

Having a blood test taken can be uncomfortable and may cause a bruise. Having spinal fluid taken may make your back hurt. However these procedures are essential whether or not you enter the study. No extra spinal fluid is taken as part of this study. The amount of extra blood taken for research tests should not affect your health. Your doctor will not do extra tests if they think it will harm you.

All samples taken will be labeled with a study number rather than your name, to protect your identity. Samples will be tested or stored indefinitely in a freezer. If you agree, further tests on stored samples may be done in the future to improve our understanding of TB meningitis. These tests may include genetic testing. We want to see whether other genes (not just the LTA4H gene) influence the way you get better from the infection. If you would like to participate in the study but do not agree to have your samples stored for later genetic testing, you can decline consent to these tests by checking the checkbox on the consent form.

**What are the drug side effects?**

All medicines can have side-effects, including the standard antibiotics given for tuberculosis treatment. Common side effects of the tuberculosis drugs include red coloration of urine (which is completely harmless and goes away when the drugs stop) and liver inflammation which can cause nausea, vomiting, and abdominal pain.

Doctors give dexamethasone for many different medical reasons and the side-effects are well known. However, when used for short periods (a few weeks, as in this trial) the side-effects of dexamethasone are generally uncommon and mild. Previous trials of dexamethasone in patients with TB meningitis have reported that side-effects are rare and it is a very safe treatment. However, side effects of dexamethasone include an increased risk of bleeding form the stomach, elevated blood sugars, nausea, confusion and an increase susceptibility to infection. If you do suffer from a side-effect that may be related to dexamethasone, the study medication will be stopped immediately and all necessary treatment will be given to you.

There is a small risk that dexamethasone may worsen hepatitis B infection. Therefore a medicine called tenofovir will also be given to patients with hepatitis B infection, to prevent worsening of the hepatitis B infection due to any immunosuppressive effect of dexamethasone.

**Participation**

Being in a research study is your decision. If you do not want to be in the study, or at any time in the study decide to stop being in the study, the doctors will respect your decision. Your medical treatment will not be affected by your decision.

If you agree to be in the study you will be responsible for following the study schedule of tests and taking your drugs. You will also be responsible for coming to the monthly visits until completing 12 months of treatment.

**Benefits and Costs**

This study may not have a direct benefit to you as it is not yet known if the study treatment will improve the treatment of patients with TB meningitis. The results from this study will help determine the best way to treat patients with this disease in the future.

You will not have to pay for any extra costs if you participate in the study. The drugs and tests which are a part of this study will be paid for by the study sponsor. If you decide to be in the study, the cost of your hospital care for tuberculosis meningitis will be covered by the study until you finish or stop participation in the study.

When you come to the hospital for follow-up visits you will be supported for your travel costs. **Confidentiality**

All information about you will be kept confidential. Your medical records will be reviewed in strict confidence by those who are working on this study and may also be reviewed by the ethics committees and health authorities reviewing the study. Your name will not be used on any of the study documents or on the stored blood samples or in any reports or publications about this study.

**Questions**

You are encouraged to ask any questions related to this study during the time of participation. If you have any other questions about the study, its procedures, risks and benefits, or alternatives please contact: {Dr. Name} at {Dr. Number}.

If you have any questions about your rights as a subject in this study, you may want to talk to {Dr. Name}, or if you want to speak to someone outside of the program you may contact the Hospital for Tropical Diseases at {Hospital Number}.

If you have general questions, please call the OUCRU Clinical Research Unit at +84 3924 1983.

Thank you for your time and your consideration to participate in this study.

**INFORMED CONSENT FORM**

**For patients enrolled in HTD only**

**Leukotriene A4 hydrolase Stratified Trial of Adjunctive Corticosteroids for HIV-uninfected adults with Tuberculous meningitis (The LAST ACT trial)**

I have read the information given to me and freely agree to be in this study. I also have had a chance to discuss it with the study staff.

I have been told about the risks and benefits. I got answers that I could understand to all my questions.

I consent to study staff collecting information about my health and using this information for future medical research.

I agree that testing on some samples may be done outside of Viet Nam.

I agree that genetic testing of the LTA4H gene will be done on my samples.

I agree that all information collected on me during the trial can be made available to others in the future (open access) provided no one can identify me from the details provided.

I understand that I can withdraw from the study at any time. If I stop the study, it will **not** affect my future care. If I decide to stop the study, I agree that the information collected up to the point when I stop, may continue to be used.

Please select whether you AGREE or DO NOT AGREE:

1.	**□ Yes or	□ No** that the samples taken from me can be stored for future testing.

2.	**□ Yes or	□ No** that the samples taken from me can be used for genetic testing

(Other than the testing of LTA4H gene mentioned above)

3.	**□ Yes or	□ No** that if my liver gets upset by the anti-tuberculosis drugs I will participate in the study of how best to manage this complication.

4.	**□ Yes or	□ No** to having brain images taken.

5.	**□ Yes or	□ No** to be in the salt and ultrasound study

6.	**□ Yes or	□ No** to be in the hormone study.

Screening Number [__][__][__][__][__]

By signing my name here, I confirm what is written above and that I have a copy of this form to keep until my part in the study ends.

| x_______________________ | x___________________ | ___/____/_____ |
| --- | --- | --- |
| Participant Signature | Print Name | Date of Signature |

**OR** – If someone else gives consent on behalf of the participant:

| Patient’s name: | x ___________________________________________ | | |
| --- | --- | --- | --- |
| Signature of Person Giving Consent:  x_______________ | Print Name:  x_____________ | Relationship to Participant:  x______________ | Date of Signature:  ____/____/_____ |

I, the undersigned, have fully explained the relevant information of this study to the person named above and will provide her/him with a copy of this signed and dated informed consent form.

| x___________________ | x_______________ | ___/____/_____ |
| --- | --- | --- |
| Investigator/Designee Signature | Print Name | Date of Signature |

**If the person giving consent cannot read the form themselves, a witness who is independent of the study must be present and sign here:**

I was present throughout the entire informed consent process with the participant. This form was read accurately to the volunteer, all questions from the volunteer were answered and the volunteer has agreed to take part in the research.

| x_______________ | x_____________________ | _____/______/_____ |
| --- | --- | --- |
| Witness Signature | Print Name | Date of Signature |
