# Supplementary material for: Adjunctive dexamethasone for the treatment of HIV-uninfected adults with tuberculous meningitis stratified by Leukotriene A4 hydrolase genotype (LAST ACT): Study protocol for a randomised double blind placebo controlled non-inferiority trial
Source: Wellcome Open Res. 2018 Mar 20;3:32. [Version 1] doi: 10.12688/wellcomeopenres.14007.1 (PMC6182672; doi:10.12688/wellcomeopenres.14007.1)
Supplement: Peer review history

# Peer review: Referee response for version 1

**DOI:** 10.21956/wellcomeopenres.15224.r34043

**Published:** 2018-10-10

**Authors:** Katalin A. Wilkinson

**Affiliations:** 1 Wellcome Centre for Infectious Diseases Research in Africa, Institute of Infectious Diseases and Molecular Medicine, University of Cape Town, Observatory, South Africa; 2 The Francis Crick Institute, London, UK

TB meningitis is the most severe form of extrapulmonary TB and adjunctive dexamethasone is currently standard of care for HIV-uninfected patients. This trial will capitalise on previous data showing that the survival benefit of adjunctive dexamethasone was restricted to the LTAH4 TT-genotype patients with high inflammatory states. Therefore, HIV uninfected TBM patients with LTAH4 CC and CT genotype will be randomised to adjunctive dexamethasone or placebo, to determine the noninferiority of placebo, with the hypothesis that dexamethasone has no benefit in patients with CC or CT genotype. The study design is complex but meticulous, with seven ancillary studies as well as a parallel, identical but independent study run in HIV-infected patients. These studies will be the ultimate proof of host directed therapies in TB.

 Questions:

1. Related to ancillary study 1 (management strategies in response to DILI): the numbers anticipated in the HIV-uninfected patients is n~70, to be analysed according to the 3 strategies. This may mean that the numbers in these analyses might be relatively low. Since the DILI strategy study will also be run in the ACT-HIV study, where anticipated numbers are n~100, will the two groups be potentially combined for analysis? It is acknowledged that this will present a number of limitations with respect to drug interactions when considering ART in the HIV-infected patients.
2. Related to ancillary study 2: will genetic variants associated with development of DILI be examined related to the host or bacteria, or both?
3. Related to ancillary study 3: the influence of dexamethasone on the resolution of inflammatory markers in the CSF will be examined. This should be done in parallel with blood, although I see no blood collection for such studies in Table 3.
4. Considering that there is no LTAH4 genotyping at weekends, and a patient recruited on a Friday may have been already on anti-TB treatment for 5 days with adjunctive dexamethasone. Therefore, by the time the genotyping result is returned on Monday with a CC genotype, the patient already received 8 days of potentially harmful dexamethasone. Assuming that this patient is randomised to placebo, the harm is potentially already done, therefore having a negative effect on the placebo arm. Is there a way to control for this, by potentially not recruiting on a Friday or introducing weekend genotyping for the duration of the trial.
5. HIV testing is done at baseline: would there be benefit in further HIV testing at 3 and/or 6 months?

I have read this submission. I believe that I have an appropriate level of expertise to confirm that it is of an acceptable scientific standard.

# Peer review: Referee response for version 1

**DOI:** 10.21956/wellcomeopenres.15224.r32233

**Published:** 2018-03-26

**Authors:** Ravindra K Garg

**Affiliations:** 1 Department of Neurology, King George's Medical University, Lucknow, Uttar Pradesh, India

This is wonderfully planned almost flawless study. I have two questions

1. Denying corticosteroids to a subgroup of patient, will it be ethically appropriate?
2.  Among   CC or CT- LTA4H genotype or TT- genotypes which one have inherently poor prognosis and need corticosteroids?  “LTA4H genotype may be a critical determinant of inflammation and consequently of adjunctive anti-inflammatory treatment response” --- outcome among varied genotypes should have been additional factor determining suitability for corticosteroids.

I have read this submission. I believe that I have an appropriate level of expertise to confirm that it is of an acceptable scientific standard.
